# Germline DNA Copy Number Aberrations Identified as Potential Prognostic Factors for Breast Cancer Recurrence

**DOI:** 10.1371/journal.pone.0053850

**Published:** 2013-01-16

**Authors:** Yadav Sapkota, Sunita Ghosh, Raymond Lai, Bradley P. Coe, Carol E. Cass, Yutaka Yasui, John R. Mackey, Sambasivarao Damaraju

**Affiliations:** 1 Cross Cancer Institute, Alberta Health Services, Edmonton, Alberta, Canada; 2 Department of Laboratory Medicine and Pathology, University of Alberta, Edmonton, Alberta, Canada; 3 Department of Oncology, University of Alberta, Edmonton, Alberta, Canada; 4 Department of Genome Sciences, University of Washington, School of Medicine, Seattle, Washington, United States of America; 5 Department of Public Health Sciences, University of Alberta, Edmonton, Alberta, Canada; The University of Texas MD Anderson Cancer Center, United States of America

## Abstract

Breast cancer recurrence (BCR) is a common treatment outcome despite curative-intent primary treatment of non-metastatic breast cancer. Currently used prognostic and predictive factors utilize tumor-based markers, and are not optimal determinants of risk of BCR. Germline-based copy number aberrations (CNAs) have not been evaluated as determinants of predisposition to experience BCR. In this study, we accessed germline DNA from 369 female breast cancer subjects who received curative-intent primary treatment following diagnosis. Of these, 155 experienced BCR and 214 did not, after a median duration of follow up after breast cancer diagnosis of 6.35 years (range = 0.60–21.78) and 8.60 years (range = 3.08–13.57), respectively. Whole genome CNA genotyping was performed on the Affymetrix SNP array 6.0 platform. CNAs were identified using the SNP-Fast Adaptive States Segmentation Technique 2 algorithm implemented in Nexus Copy Number 6.0. Six samples were removed due to poor quality scores, leaving 363 samples for further analysis. We identified 18,561 CNAs with ≥1 kb as a predefined cut-off for observed aberrations. Univariate survival analyses (log-rank tests) identified seven CNAs (two copy number gains and five copy neutral-loss of heterozygosities, CN-LOHs) showing significant differences (*P*<2.01×10^−5^) in recurrence-free survival (RFS) probabilities with and without CNAs.We also observed three additional but distinct CN-LOHs showing significant differences in RFS probabilities (*P*<2.86×10^−5^) when analyses were restricted to stratified cases (luminal A, n = 208) only. After adjusting for tumor stage and grade in multivariate analyses (Cox proportional hazards models), all the CNAs remained strongly associated with the phenotype of BCR. Of these, we confirmed three CNAs at 17q11.2, 11q13.1 and 6q24.1 in representative samples using independent genotyping platforms. Our results suggest further investigations on the potential use of germline DNA variations as prognostic markers in cancer-associated phenotypes.

## Introduction

Breast cancer is the most common epithelial malignancy among women in the developed world, with more than 200,000 new cases and 39,000 deaths estimated in the United States in 2012 [1]; comparable statistics were also observed in Canada in 2011 [2]. While age-adjusted breast cancer incidence has increased with the introduction of screening measures, there has been a steady decline in breast cancer mortality rates over the last two decades. During the years 1998–2008, cancer related death rates have decreased by more than 1% per year in North American women and breast cancer explains one-third of this total decline [1].

Advances in early diagnosis, increased public awareness and improved adjuvant treatment modalities have contributed to the improvements in prognosis of early-stage breast cancer. Standard guideline-based therapy for non-metastatic breast cancer typically includes surgical excision of localized tumor and involved lymph nodes, followed by adjuvant systemic and radiotherapies to eradicate any residual micro-metastatic deposits. Both systemic chemotherapy and adjuvant endocrine therapy have reduced breast cancer recurrence and death [3]. However, currently used adjuvant therapies have life-threatening and life-altering toxicities, and it therefore is of clinical importance to identify patients who would most benefit from aggressive adjuvant therapies, and to spare those patients unlikely to benefit from aggressive therapy. At present, the determination of those breast cancer patients who are most likely to benefit from adjuvant therapies is primarily guided by tumor-based prognostic factors such as axillary lymph nodal status, tumor size, tumor histologic grade, lymphatic and vascular invasion, proliferative markers, ER/progesterone receptor (PR) and human epidermal growth factor receptor 2 (HER2) status [4], [5]. However, clinicopathological characteristics of tumors remain imperfect prognostic classifiers, in part due to the molecular heterogeneity of breast cancer.

While genomic signatures derived from tumor transcriptome studies such as 21-gene and 70-gene profiles may provide some improvement in prognostic power when added to standard clinicopathologic prognosticators, there are still patients who experience recurrence who are categorized as having an excellent prognosis, and others who remain recurrence free who are categorized as having a very poor prognosis [6], [7]. Furthermore, despite incremental improvement in breast cancer therapies, approximately 30% of the treated breast cancer patients (who are non-metastatic at the time of diagnosis) show disease recurrence within ten years [8], [9]. Consequently, there remains continued need to identify improved prognostic and predictive markers with higher performance for clinical validation in prospective studies.

Recent studies show that germline DNA variations contribute to disease susceptibility [10]–[12], prognosis [13]–[15] and response to therapies [16], [17]. The majority of these studies have adopted widely accepted multi-stage association study designs using single nucleotide polymorphisms (SNPs) from candidate genes/pathways or whole genome scans. As a result, thousands of SNPs have been identified that are significantly associated with susceptibility to breast cancer and its subtypes [12], [18], [19] and some of these are likely to predict overall disease survival [13]–[15]. In addition to SNPs, germline copy number variations (CNVs) are also found to be an important source of genetic predisposition to many complex phenotypes, including breast cancer [20]–[24]. CNVs are the most common type of genetic structural variations and by definition show gains or losses of DNA segments comprising more than one kb [21], [22]. These DNA variations are believed to exert their affects through gene expression either through gene-dosage or *cis*-acting gene regulatory activities [25], [26]. More recently with the application of high-throughput SNP-arrays, large chromosomal lesions characterized by loss of heterozygosity (LOH) but with diploid copy number were observed in many tumor types, possibly resulting from mitotic recombination [27]–[29]. These unique regions are referred to as copy neutral-loss of heterozygosities (CN-LOHs) or uniparental disomies (UPDs). Interestingly, large CN-LOH regions were also found in germline DNA and these genomic signatures may also be of value as potential markers for susceptibility and prognosis of complex diseases, such as cancers [30]–[33].

In the present study, we analyzed germline CNVs and CN-LOHs (hereafter referred to as copy number aberrations, CNAs) genotyped with Affymetrix Genome-Wide Human SNP Array 6.0 (Santa Clara, CA, USA) for their role as potential prognostic markers using 369 breast cancer patients from Alberta, Canada, treated with standard guideline-based therapies and followed over extended periods to capture the disease recurrence. We confirmed select CNAs identified from Affymetrix SNP 6.0 array data by independent technology platforms; TaqMan real-time quantitative polymerase chain reaction (RT-qPCR) (Carlsbad, CA, USA) for copy number determination and Sequenom iPLEX Gold Platform (San Diego, CA, USA) for assessing the fraction of heterozygosity in a subset of samples, using services from the McGill University, Genome Quebec Innovation Center, Montreal, Canada.

## Materials and Methods

### Patients

Breast cancer cases were accessed from the PolyomX and Canadian Breast Cancer Foundation (CBCF) Tumor Banks, located at the Cross Cancer Institute, Edmonton, Alberta, Canada [10], [11]. The subject recruitment criteria and geographic populations of the PolyomX Tumor Bank and its successor, the CBCF Tumor Bank, (accrual during 2001–2005 and 2005-present, respectively) were the same. These tumor banks contain flash frozen tumor specimens, matching buffy coat samples (from over 2,000 subjects, diagnosed between the years 1987 to 2012) and clinicopathological information for breast and other cancers in the province of Alberta (http://www.abtumorbank.com/). In this study, we included 369 Caucasian women (median age = 51 years) with a confirmed diagnosis of early-stage non-metastatic breast cancer predominantly characterized by late onset of disease and with the criteria identified below for case selection. Despite standard adjuvant therapy, 155 patients (median follow-up time from diagnosis = 6.30 years; range = 0.60–21.78 years) experienced recurrence and 214 did not, after a minimum duration of follow up of three years (median follow-up time from diagnosis = 8.60 years; range = 3.08–13.57 years). Of the 214 cases, follow-up time for (i) 32 (14.95%) was between three to five years, (ii) 40 (18.69%) was between five to seven years, (iii) 105 (49.06%) was between seven to ten years and (iv) 37 (17.29%) was more than ten years.

Of 369 individuals, 286 (77.50%) were ≥45 years old. Following diagnosis, these women received curative-intent primary treatments (surgical resection, chemotherapy with anthracyclines and/or taxanes, trastuzumab, hormonal therapy and radiotherapy) as per standardized provincial breast cancer care. A detailed description of clinicopathological characteristics of breast cancer patients is presented in Table 1, and the outcome data reflects database updates up to 21^st^ February 2012. Informed consents were obtained from all study participants and the study was approved by the Research Ethics Board of Alberta Health Services.

**Table 1:** Clinicopathological characteristics of 369 breast cancer cases enrolled in the study.

Characteristics	BCR (n = 155)	non-BCR (n = 214)	*P* value^a^	
Median age at diagnosis (yrs.)	51	51.5	0.90	
Follow-up time from diagnosis (days)^b^	2,317 (219–7948)	3,138 (1125–4954)		
Molecular subtypes			**0.01**	
* Luminal A*	82	129		
* Luminal B*	25	37		
* HER2 type*	11	14		
* Triple negative*	28	14		
* Other(s)*	9	20		
Menopausal status			0.20	
* Pre*	62	80		
* Peri*	19	17		
* Post*	73	117		
* Unknown*	1	0		
Family history of breast cancer			0.37	
* Yes*	59	95		
* No*	91	115		
* Unknown*	5	4		
Overall grade			**8.2×10^−4^**	
* Low*	63	126		
* High*	89	87		
* Unknown*	3	1		
Stage			**0.01**	
* I*	21	38		
* II*	106	159		
* III*	28	17		

a *P* values for Median age at diagnosis (yrs.) was calculated using Mann-Whitney test whereas 2×n Fisher's exact test was used for Molecular subtypes, Menopausal status, Family history of breast cancer, Overall grade and Stage.

b Median is presented with range shown in the parentheses.

*P* values<0.05 is indicated in bold.

Breast cancer patients enrolled in the study were further classified into tumor subtypes based on immunohistochemistry score-based ER, PR and HER2 status of tumors as recorded in pathology reports. Using conventional guidelines commonly used in epidemiological studies [34], tumors were categorized as (i) luminal A for ER^+^ and/or PR^+^ and HER2^−^, (ii) luminal B for ER^+^ and/or PR^+^ and HER2^+^, (iii) HER2 type for ER^−^, PR^−^ and HER2^+^, (iv) triple negative for ER^−^, PR^−^ and HER2^−^. There were 211 luminal A cases (170 with ER^+^ and/or PR^+^ and HER2^−^ and 41 with both ER^+^ and PR^+^ and unknown HER2 status but characterized by low tumor grade). Among the remaining cases, there were 62 luminal B, 25 HER2 type, and 42 triple negative cases. There were 29 cases with unknown HER2 status and varying combinations of ER and PR (+ or – status) and tumor grades (high or low) that were, therefore, classified as others and excluded from the finer analyses based on stratification of the molecular subtypes of breast cancer. We adhered to the Recommendations for Tumor Marker Prognostic Studies (REMARK) [35] for the results reported, where applicable.

### DNA Extraction, Whole Genome Genotyping and Quality Control

DNA was extracted from the buffy coat fractions using commercially available Qiagen™ (Mississauga, Ontario, Canada) DNA isolation kits. Buffy coat fractions collected were stored at -80 °C until use. Following guidelines provided by manufacturer, whole genome genotyping was conducted using Affymetrix Genome-Wide Human SNP Array 6.0, which consisted of over 1.8 million probes (906,600 SNPs and 946,000 copy number probes) with an overall inter-marker distance of 680 bp. We used Affymetrix recommended contrast quality control (CQC), a measure of performance of genotyping experiments, to assess sample quality. All 369 samples used in this study showed CQC>2.0, a value greater than the default CQC threshold of ≥1.7.

### Identification of CNAs

We used Nexus Copy Number 6.0 genomics software to process Affymetrix generated signal intensity or CEL files. A reference genome created using 270 HapMap samples was used as a baseline to calculate log2ratios and B-allele frequencies (BAF) in each sample followed by quantile normalization [36]. Probe to probe variance was calculated and reported as quality control (QC) scores to remove extreme outliers due to copy number break-points. We used a default setting for outlier removal, a combined value of 3% at the two extremes, 1.5% at each end. Using these normalized log2ratio and BAF values, CNAswere identified with the SNP-Fast Adaptive States Segmentation Technique 2 (SNP-FASST2) segmentation algorithm in conjunction with quadratic wave correction implemented in the Nexus software. The SNP-FASST2 segmentation algorithm is a Hidden Markov Model-based approach, which uses log2ratio values of ∼1.8 million probes to make a CNV call while it considers both log2ratio and BAF values to detect LOHs. Significance threshold for segmentation was set at *P*<5×10^−7^with minimum number of ten probes per segment and a maximum probe spacing of 1,000 kb. Single copy gains and losses were defined with log2ratio values of 0.2 and -0.2, respectively while two or more than two copies of gains and losses were defined by log2ratio values of 0.7 and -1.1, respectively. A chromosomal region was called a LOH if ≥95% of the SNP probes in a DNA segment of at least 500 kb exhibited BAF≥0.8 or ≤0.2– i.e., ≥95% of the SNP probes in that region are homozygous probes (*e.g.*, AA or BB). Auto gender correction available in Nexus software was applied to call CNAs in X chromosomes. LOHs with diploid copy number of two were considered as CN-LOHs or UPDs.

### Quality Control Parameters for CNA Calling

Pre-processing of CEL files was conducted using the settings described above. Six (three luminal A, two luminal B and one HER2 type tumors) out of 369 samples exhibited very high QC scores (>0.40) and were excluded from final analyses as higher QC scores suggest for elevated noise to signal ratio. Average QC score of remaining 363 samples (152BCR and 211 non-BCR) was 0.17 (range: 0.08–0.32), acceptable values recommended by the Nexus Copy Number 6.0.

### Survival Analysis of CNAs and Statistical Considerations

Of the CNAs identified by the SNP-FASST2 segmentation algorithm, we restricted our analysis to relatively high frequency common CNAs to evaluate their potential role in breast cancer recurrence because common CNVs often harbour cancer-related genes [37]. We excluded LOHs due to copy number losses and more than two copy number gains from the analysis as these were already captured as copy number losses and copy number gains, respectively. We used a frequency cut-off of ≥10% in either group (BCR and non-BCR) or in both to select relatively common CNAs in our study population. When overlaps between CNAs selected in BCR and non-BCR groups were noted, we considered the intersecting common CNA regions present in both groups.

Univariate survival analyses showing relationships between select germline CNAs and recurrence-free survival (RFS) were performed using Kaplan-Meier survival curves. RFS probabilities with and without CNAs in 363 samples were estimated using log-rank tests with one degree of freedom (d.f.). Correction for multiple hypotheses testing was carried out using the Benjamini-Hochberg False Discovery Rate correction method and represented as *Q* value [38]. Association of germline CNAs with BCR was determined with univariate Cox proportional hazards model and reported as hazard ratios (HRs) and corresponding 95% confidence intervals (CIs). Tumor stage and grade were then included as covariates in the Cox proportional hazards model to estimate the adjusted HRs and corresponding 95% CIs.

We also conducted subgroup survival analyses (log-rank tests with one d.f.) to identify additional common CNAs specific to luminal A subtype of breast cancer wherein we compared RFS probabilities with and without CNAs in 208 luminal A samples only. CNAs for association testing were selected using the approach mentioned above (*i.e*., we focused on relatively common CNAs with ≥10% frequencies in at least one group or in both). Association analyses per se were carried out by fitting Cox proportional hazards models as explained earlier. Subgroup analyses restricted to luminal B, HER2 type and triple negative samples were not attempted due to limited sample size.

All statistical analyses were carried out, either singly or in combination using R 2.14.1 (R Development Core Team, 2011) and SAS software, version 9.3 of the SAS system for Windows. Copyright© 2002–2010 SAS Institute Inc. Cary, NC, USA.

### Validation of Candidate CNAs Using Independent Genotyping Platforms

Potential candidate CNAs were validated in a representative subset of samples. Using services from the McGill University, Genome Quebec Innovation Center, Montreal, Canada, we quantified the copy number of candidate CNAs using pre-designed TaqMan® copy number assays on a RT-qPCR instrument (Applied Biosystems, Foster City, CA, USA). Primers and probes targeted for individual copy number assays were from within the candidate CNA sequence boundaries identified in Nexus. We used 2 µL per assay of genomic DNA at a final concentration of 20 ng/µL. All reactions were run in quadruplicates in MicroAmp® optical 96-well plates with barcode sealed with optical adhesive film. Thermal-cycling (7900HT) conditions were: 10 minutes at 95°C followed by 40 cycles of 15 seconds at 95°C and 60 seconds at 60°C. Real-time data was exported to CopyCaller v2.0. RNaseP was used as a reference to calculate the ΔCt values for each sample. Copy numbers were determined using the comparative ΔΔ^C^t cycle threshold method, assuming most frequent sample copy number of two. For CN-LOHs, SNPs (ten per CN-LOH) were also genotyped for same DNAs used in copy number assays using the Sequenom iPLEX Gold Platform to measure percentages of heterozygosity in CN-LOHs. Using HapMap release 24 Central Europeans genotype data, tagSNPs for CN-LOHs were selected with minor allele frequency (MAF) and pair-wise correlation (*r^2^*) cut-offs of 10% and 0.8, respectively, to ensure the large CN-LOH region SNPs selected were non redundant. Whenever number of tagSNPs was less than ten, additional SNPs with ≥10% global MAF (1000 Genomes Project phase 1 population of 629 individuals) from NCBI dbSNP build 136 were randomly selected ensuring that none of these additional SNPs was tagged by previously selected tagSNPs (see Table S1 for probe selection and relevant assays).

## Results

### Patients’ Clinical Characteristics

We identified 369 cases as meeting the criteria for the study of BCR and non-BCR, as described in the methods. We investigated if the clinical characteristics for study subjects (BCR and non-BCR cases) were different and how these might contribute to potential confounding effects. We did not find statistically significant differences for age at diagnosis, menopausal status and family history of breast cancer between BCR and non-BCR while molecular subtypes, tumor overall grade and stage were significantly different between BCR and non-BCR (Table 1). The identified potential confounders were taken into consideration for the data analysis and interpretations.

### Summary of CNAs Identified

**Figure 1:**
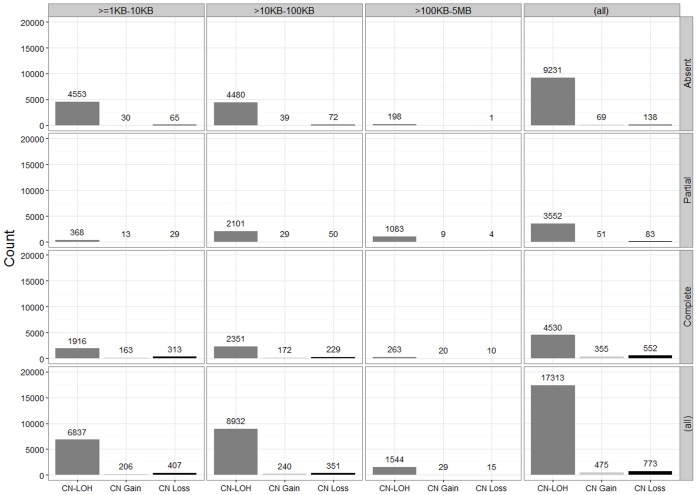
Absolute counts of CNAs stratified by overlap with germline CNVs in DGV and their length. Shown in the histograms are total numbers of copy number losses (CN Loss), copy number gains (CN Gain) and copy neutral-loss of heterozygosities (CN-LOHs) identified in 363 samples stratified by their lengths (≥1 KB–10 KB, >10 KB–100 KB and >100 KB–5 MB) and their overlap with known germline CNVs in the Database of Genomic Variants (DGV), Toronto. A 100% overlap is shown as ‘Complete’, less than 100% but more than 0% is shown as ‘Partial’ and no overlap is shown as ‘Absent’.

SNP-FASST2 algorithm identified 19,591CNAs (516 copy number gains, 869 copy number losses and 18,206 CN-LOHs) in 363 samples (Table S2). Of these, 18,561 CNAs (475 copy number gains, 773 copy number losses and 17,313 CN-LOHs) were of ≥one kb (Figure 1).Majority of copy number gains (n = 465), copy number losses (n = 746) and CN-LOH (n = 15,682) were in chromosomes 1 to 22 while very few events (10 copy number gains, 27 copy number losses and 1,631 CN-LOHs) were observed in X-chromosomes. A total of 7,450CNAs were of >1 kb–10 kb, 9,523 CNAs were of >10 kb–100 kb and 1,588 CNAs were very large regions (>100 kb–5 Mb). We observed three copy number gains (two in chromosome 14 and one in chromosome 2) that were present in all 363 samples. Moreover, 9,123 (approximately 50%) of the CNAs identified in our 363 samples exhibited either complete (100%) or partial overlap (more than 0% but less than 100%) with known germline CNVs reported in the Database of Genomic Variants (DGV), Toronto (http://projects.tcag.ca/variation/). There were 9,438 (69 copy number gains, 138 copy number losses and 9,231 CN-LOHs) observed in the current study that are absent in the DGV (0% overlap) and hence may be novel chromosomal aberrations that merit independent replication.

### CNAs Associated with BCR

**Figure 2:**
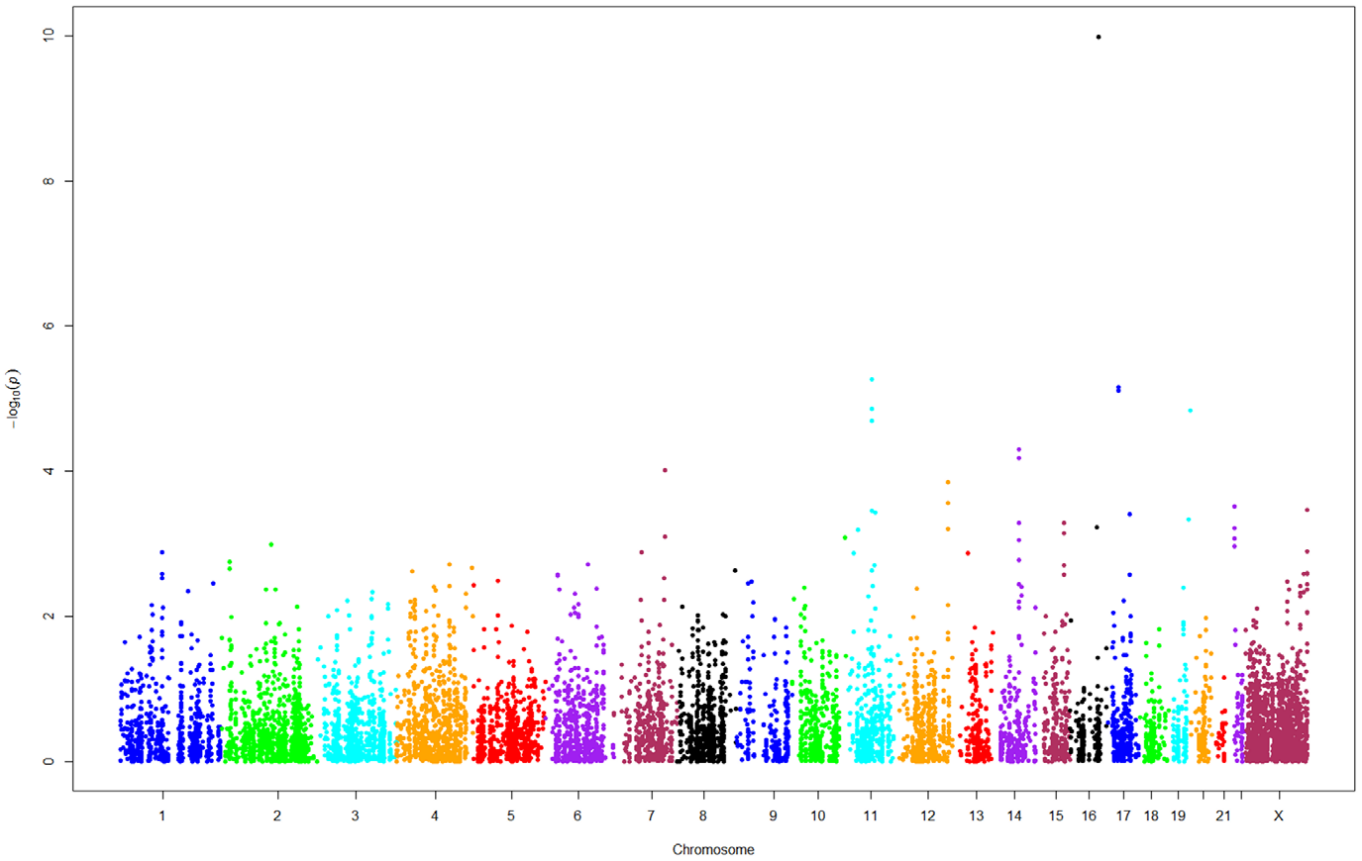
Chromosome-wide distributions of 9,164 CNAs tested for association with BCR in unstratified samples. Shown on the x-axis are middle points of chromosomal start and end positions (NCBI Build 37) of 9,161CNAs and on the y-axis are –log10 *P* values for their association with the phenotype of BCR in unstratified 363 samples. *P* values were obtained from log-rank tests with one d.f.

Of the 18,561 CNAs with more than one kb (152 BCR and 211 non-BCR), we found 9,164 CNAs (145 copy number gains, 241 copy number losses and 8,778 CN-LOHs) with ≥10% frequency either in the BCR or non-BCR groups or in both. When we compared RFS probabilities with and without these CNAs in 363 samples, we found that 585 CNAs (33 copy number gains, 33 copy number losses and 519 CN-LOHs) showed statistically significant differences in RFS probabilities at nominal *P*<0.05 (Figure 2). Of these, two copy number gains and five CN-LOHs showed the strongest differences in RFS probabilities (*P*<2.01×10^−5^, *Q*<0.03) (Figure 2, Table 2) and all seven CNAs (three CNAs at chromosome 11, two at chromosome 17 and one CNA each at chromosomes 16 and 19) were also associated with increased risk of recurrence.

**Table 2:** Chromosomal aberrations statistically significantly associated with BCR in 363 samples.

Chromosomal regions^a^	Cytoband	Length (bps)	Event	Overlap with DGV	Genes/loci	No. of events	*P* value^b^	Q value^c^	HR_unadusted_, 95% CI	HR_adjusted_ d, 95% CI
chr16∶70151941-70198049	q22.1	46,109	CN Gain	Complete	*CLEC18A/PDPR*	21	1.02×10^−10^	9.35×10^−7^	4.49 [2.73–7.40]	3.88 [2.35–6.41]
chr17∶30556456-30568424	q11.2	11,969	CN-LOH	Absent	Intergenic	66	7.79×10^−6^	0.02	2.20 [1.54–3.13]	2.09 [1.46–2.99]
chr11∶64169509-64201009	q13.1	31,501	CN-LOH	Absent	Intergenic	23	5.46×10^−6^	0.02	2.98 [1.82–4.88]	2.28 [1.35–3.85]
chr17∶30292345-30436095	q11.2	143,751	CN-LOH	Complete	*SUZ12/LRRC37B/SH3GL1P1*	68	6.90×10^−6^	0.02	2.19 [1.54–3.12]	2.06 [1.45–2.94]
chr19∶53519960-53538651	q13.41	18,692	CN Gain	Complete	Intergenic	47	1.44×10^−5^	0.02	2.35 [1.58–3.50]	2.34 [1.56–3.51]
chr11∶64228316-64258125	q13.1	29,810	CN-LOH	Absent	Intergenic	36	1.39×10^−5^	0.02	2.52 [1.64–3.88]	2.08 [1.32–3.26]
chr11∶64048319-64143935	q13.1	95,617	CN-LOH	Partial	*BAD/KCNK4/* *GPR137/ESRRA/* *CCDC88B*	25	2.01×10^−5^	0.03	2.74 [1.69–4.43]	2.15 [1.29–3.58]

a chromosomal positions are based on NCBI build 37; DGV, Database of Genomic Variants (Toronto); HR, hazard ratio; CI, confidence interval;

b *P* value obtained from log-rank test with one d.f.;

c FDR corrected for multiple hypothesis testing;

d adjusted for tumor stage and grade; *CLEC18A*, C-type lectin domain family 18, member A; *PDPR*, pyruvate dehydrogenase phosphatase regulatory subunit; *SUZ12*, suppressor of zeste 12 homolog (*Drosophila*); *LRRC37B*, leucine rich repeat containing 37B; *SH3GL1P1*, SH3-domain GRB2-like 1 pseudogene 1; *BAD*, BCL2-associated agonist of cell death; *KCNK4*, potassium channel, subfamily K, member 4; *GPR137*, G protein-coupled receptor 137; *ESRRA*, estrogen-related receptor alpha; *CCDC88B*, coiled-coil domain containing 88B.

#### Chromosome 11 CNAs

(i) A CN-LOH of 31,501 bp at chromosome 11q13.1 indicating significant differences in RFS probabilities (*P* = 5.46×10^−6^, *Q* = 0.02) was associated with BCR (HR_unadjusted_, 95% CI = 2.98 [1.82–4.88]; HR_adjusted_, 95% CI = 2.28 [1.35–3.85]). We did not observe any germline CNVs in the DGV overlapping with this CNA. (ii) A CN-LOH of 29,810 bp at chromosome 11q13.1 indicating significant differences in RFS probabilities (*P* = 1.39×10^−5^, *Q* = 0.02) was associated with BCR (HR_unadjusted_, 95% CI = 2.52 [1.64–3.88]; HR_adjusted_, 95% CI = 2.08 [1.32–3.26]). There were no overlapping known CNVs reported in the DGV. (iii) Another CN-LOH of 95,617 bp at chromosome 11q13.1 (exhibiting partial overlap with germline CNVs in DGV) showing significant differences in RFS probabilities (*P* = 2.01×10^−5^, *Q* = 0.03) was associated with BCR (HR_unadjusted_, 95% CI = 2.74 [1.69–4.43]; HR_adjusted_, 95% CI = 2.15 [1.29–3.58]).

#### Chromosome 17 CNAs

(i) A CN-LOH of 11,969 bp at chromosome 17q11.2 showing significant differences in RFS probabilities (*P* = 7.79×10^−6^, *Q* = 0.02) was associated with BCR (HR_unadjusted_, 95% CI = 2.20 [1.54–3.13]; HR_adjusted_, 95% CI = 2.09 [1.46–2.99]). We did not find any known germline CNVs in the DGV that overlapped with this CN-LOH, suggesting that it could be a novel CNA. (ii) A CN-LOH of 143,751 bp at chromosome 17q11.2 (exhibiting complete overlap with germline CNVs in DGV) with significant differences in RFS probabilities (*P* = 6.90×10^−6^, *Q* = 0.02) was also associated with BCR (HR_unadjusted_, 95% CI = 2.19 [1.54–3.12]; HR_adjusted_, 95% CI = 2.06 [1.45–2.94]).

#### Chromosome 16 CAN

A copy number gain of 46,109 bp at chromosome 16q22.1 (with complete overlap with germline CNVs in DGV) that showed significant differences in RFS probabilities (*P* = 1.02×10^−10^, *Q* = 9.35×10^−7^) was associated with BCR (HR_unadjusted_, 95% CI = 4.49 [2.73–7.40]; HR_adjusted_, 95% CI = 3.88 [2.35–6.41]).

#### Chromosome 19 CAN

A copy number gain of 18,692 bp at chromosome 19q13.41 (with complete overlap with germline CNVs in DGV) showing significant differences in RFS probabilities (*P* = 1.44×10^−5^, *Q* = 0.02) was associated with BCR (HR_unadjusted_, 95% CI = 2.35 [1.58–3.50]; HR_adjusted_, 95% CI = 2.34 [1.56–3.51]).

We then compared the RFS probabilities of the above seven CNAs(i.e., with similar start and end positions) in each of the molecular subtypes of breast cancer using log-rank tests with one d.f. to examine for possible overlap of these genomic signatures across molecular subtypes. Differences in RFS probabilities and magnitude and direction of associations (HRs and corresponding 95% CIs) of all seven CNAs with BCR in 208 luminal A samples (80 BCR and 128 non-BCR) were comparable to those observed in entire 363 samples (Table 3). However, the differences in RFS probabilities were statistically non-significant in other subtypes (luminal B, HER2 type and triple negative), except for a copy number gain at chromosome 16q22.1 (*P*<6.15×10^−3^) in luminal B and triple negative subtypes, for a CN-LOH at chromosome 17q11.2 (*P* = 2.63×10^−3^) in the luminal B subtype and for a CN-LOH at chromosome 11q13.1 (*P* = 5.35×10^−4^) in the triple negative subtype (Table S3). Thus, the seven CNAs reported here appeared to be relatively specific to the luminal A subtype of breast cancer, as would be expected of the sample composition with luminal A cases as a major subset.

**Table 3:** Association of top seven CNAs (Table 2) with BCR in 208 luminal A samples.

Chromosomal regions^a^	Event	No. of events	*P* value	HR_unadjusted_, 95% CI	HR_adjusted_ b, 95% CI
chr16∶70151941-70198049	CN Gain	10	2.95×10^−6^	4.92 [2.35–10.33]	4.95 [2.30–10.67]
chr17∶30556456-30568424	CN-LOH	39	8.13×10^−4^	2.23 [1.38–3.60]	2.01 [1.22–3.29]
chr11∶64169509-64201009	CN-LOH	12	9.08×10^−4^	3.07 [1.53–6.17]	1.89 [0.89–4.05]
chr17∶30292345-30436095	CN-LOH	39	6.70×10^−4^	2.26 [1.39–3.65]	1.98 [1.21–3.25]
chr19∶53519960-53538651	CN Gain	24	1.83×10^−7^	3.75 [2.20–6.39]	4.08 [2.29–7.26]
chr11∶64228316-64258125	CN-LOH	20	3.25×10^−7^	3.84 [2.20–6.69]	2.82 [1.54–5.14]
chr11∶64048319-64143935	CN-LOH	13	3.89×10^−4^	3.15 [1.61–6.14]	2.03 [0.98–4.19]

a chromosomal positions are based on NCBI build 37;

b adjusted for tumor stage and grade.

### Subgroup Analysis Restricted to Luminal A Samples (n = 208)

**Figure 3:**
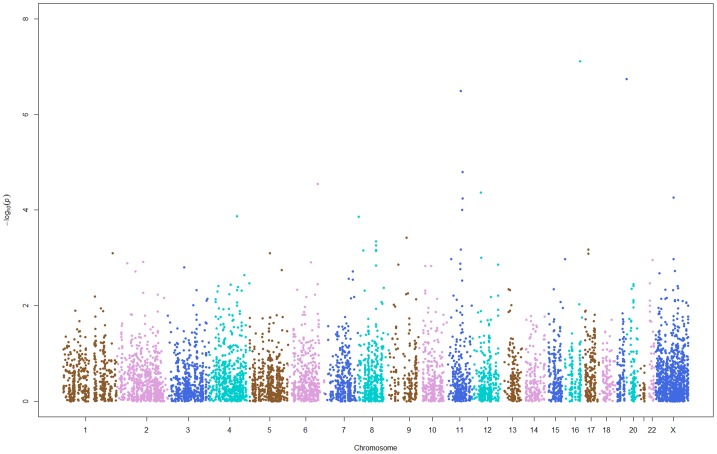
Chromosome-wide distributions of 7,218 CNAs tested for association with BCR in 208 luminal A cases. Shown on the x-axis are middle points of chromosomal start and end positions (NCBI Build 37) of 7,218 CNAs and on the y-axis are –log10 *P* values for their association with the phenotype of BCR in 208 luminal A cases. *P* values were obtained from log-rank tests with one d.f.

In an attempt to identify additional CNAs, we estimated the differences in RFS probabilities with and without CNAs in the luminal A subtype (80 BCR and 128 non-BCR) of breast cancer. We identified 7,218 CNAs (142 copy number gains, 258 copy number losses and 6,818 CN-LOHs) with ≥10% frequency either in at least one group or in both. Of these, 4,379 CNAs shared commonality with 9,164 CNAs observed in the entire 363 samples while 2,839 CNAs were distinct, owing to the variant start and end positions, chromosomal locations or the indicated frequency threshold of ≥10% in BCR or non-BCR cases or in both. We identified a total of 484 of 7,218 CNAs (27 copy number gains, 32 copy number losses and 425 CN-LOHs) showing statistically significant differences in RFS probabilities with and without CNAs at nominal *P*<0.05 (Figure 3). Of these, three CN-LOHs showed the strongest statistically significant differences in RFS probabilities in the luminal A subtype of breast cancer (Table 4), vis-à-vis from the 2,839 distinct CNAs in this sub group.

**Table 4:** Additional CNAs statistically significantly associated with BCR in 208 luminal A samples.

Chromosomal regions^a^	Cytoband	Length (bp)	Event	Overlap with DGV	Genes/loci	No. of events	*P* value^b^	Q value^c^	HR_unadjusted_, 95% CI	HR_adjusted_ d, 95% CI
chr11∶64228316-64285905	q13.1	57,590	CN-LOH	Absent	Intergenic	20	3.25×10^−7^	7.82×10^−4^	3.84 [2.20–6.69]	2.82 [1.54–5.14]
chr11∶72198387-72208236	q13.4	9,850	CN-LOH	Absent	Intergenic	16	1.62×10^−5^	0.03	3.58 [1.93–6.66]	2.60 [1.27–5.31]
chr6∶140631638-140723307	q24.1	91,670	CN-LOH	Absent	Intergenic	52	2.86×10^−5^	0.04	2.54 [1.62–3.98]	2.38 [1.50–3.76]

a chromosomal positions are based on NCBI build 37; DGV, Database of Genomic Variants (Toronto); HR, hazard ratio; CI, confidence interval;

b *P* value obtained from log-rank test with one d.f.;

c FDR corrected for multiple hypothesis testing;

d adjusted for tumor stage and grade.

The three distinct CNAs identified in this sub group showed the following characteristics. (i) A CN-LOH of 57,590 bp at chromosome 11q13.1 showing significant differences in RFS probabilities (*P* = 3.25×10^−7^, *Q* = 7.82×10^−4^) was associated with increased risk of BCR (HR_unadjusted_, 95% CI = 3.84 [2.20–6.69]; HR_adjusted_, 95% CI = 2.82 [1.54–5.14]). (ii) A CN-LOH of length 9,850 bp at chromosome 11q13.4 indicating significant differences in RFS probabilities (*P* = 1.62×10^−5^, *Q* = 0.03) was associated with increased risk of BCR (HR_unadjusted_, 95% CI = 3.58 [1.93–6.66]; HR_adjusted_, 95% CI = 2.60 [1.27–5.31]). (iii) And lastly, a CN-LOH of length 91,670 bp at chromosome 6q24.1 showing significant differences in RFS probabilities (*P* = 2.86×10^−5^, *Q* = 0.04) was associated with increased risk of BCR (HR_unadjusted_, 95% CI = 2.54 [1.62–3.98]; HR_adjusted_, 95% CI = 2.38 [1.50–3.76]). We did not find any known germline CNVs in the DGV that overlapped with these three CN-LOHs.

We did not find statistically significant differences in RFS probabilities with and without above three CN-LOHs (11q13.1, 11q13.4 and 6q24.1) in luminal B, HER2 type or triple negative subtypes suggesting that these CN-LOHs were relatively specific to the luminal A subtype of breast cancer (Table S3). Overall, chromosome 11 appears to harbour multiple CN-LOHs (identified 5 CNAs in total, Tables 2, 3, and 4 and Table S3) and these showed increased risk of BCR in the luminal A subtype of breast cancer.

Moreover, adjustment in HRs and 95% CI for tumor grade and stage in the analyses presented thus far revealed minimal or no evidence of potential confounding effects. Hence, these clinicopathological characteristics are less likely to significantly modify the observed associations of identified CNAs with BCR at the indicated sample size (Tables 1–4).

### RT-qPCR Validation of Select CNAs in Representative Samples

Of the ten CNAs (eight CN-LOHs and two copy number gains) showing statistically significant association with the phenotype of BCR, we chose to validate three relatively longer CN-LOHs (143,751 bp at 17q11.2, 57,590 bp at 11q13.1 and 91,670 bp at 6q24.1) in a subset of 363 samples (a combination of randomly selected samples harbouring the CN-LOHs plus approximately equal proportion of samples without these CN-LOHs as evaluated by the Nexus Copy Number 6.0) by RT-qPCR and Sequenom genotyping. There is a growing consensus that CN-LOHs are important in the genomes profiled using germline DNA in the recent literature [27]–[29], [32] and this formed the basis for validating the most predominant chromosomal aberrations in this study. These newly emerging chromosomal aberrations (CN-LOHs), if confirmed, may be included in future investigations alongside the copy loss or gain aberrations for a comprehensive catalogue of CNAs relevant for complex/polygenic phenotypes. Remaining two CN-LOHs were shorter in size and were not considered for validation. First, we quantified the copy number status of these three CN-LOHs using copy number assays (Hs00138078_cn and Hs02495547_cn for CN-LOH at 17q11.2 in 38 samples (interrogated using two assays in this region, largest of the CN-LOH identified in this study), Hs06324464_cn for CN-LOH at 11q13.1 in 33 samples and Hs06809880_cn for CN-LOH at 6q24.1 in 36 samples). Concordance between copy number calls made from Nexus read-out and RT-qPCR was 100% showing copy number of two (Table S4). Second, we successfully genotyped 24 of 30 SNPs initially selected for three CN-LOHs in the same DNA samples used for copy number assays to measure percentage of heterozygosity in each CN-LOH; assays of six SNPs were not successful. Percentages of heterozygosity were calculated using seven SNPs for CN-LOH at 17q11.2, nine SNPs for CN-LOH at 11q13.1 and eight SNPs for CN-LOH at 6q24.1. Pearson correlations coefficients between heterozygote frequencies measured from Affymetrix SNP 6.0 array data and from Sequenom iPLEX Gold Platform for CN-LOH at 17q11.2, CN-LOH at 11q13.1 and CN-LOH at 6q24.1 were 0.97, 0.98 and 0.99, respectively (Table S4).

## Discussion and Conclusion

In this study, we identified ten germline CNAs as potential prognostic factors for disease recurrence in the early-stage non-metastatic breast cancer. These germline signatures were particularly relevant to the luminal A subtype as large number of breast cancer cases with luminal A tumors experience disease recurrence despite their good prognosis based on tumor characteristics. Using a sample size of 363 breast cancer patients who received standard guideline-based therapy upon diagnosis, we demonstrated statistically significant associations of ten CNAs (two copy number gains and eight CN-LOHs) with the phenotype of BCR in both univariate and multivariate analyses (adjusted for tumor stage and grade). Three CN-LOHs (17q11.2, 11q13.1 and 6q24.1) were validated in a subset of 363 samples using RT-qPCR and Sequenom iPLEX Gold Platform technologies. Adjustment for tumor stage and grade did not influence the direction or effect size reported in terms of the HRs and 95% CI, suggesting that these clinicopathological characteristics did not influence the observed association results. As such, these germline CNAs may offer significant prognostic value for breast cancer, independent of tumor clinicopathological characteristics considered in this study.

While many studies have evaluated potential role of copy number gains, copy number losses and classical LOHs, only a few have investigated the impact of CN-LOH in complex diseases such as cancer [27]–[29]. This may be due to inadequate karyotyping technology as conventional cytogenetics (array-CGH) and fluorescence in situ hybridization (FISH) cannot detect these small unique chromosomal aberrations. However, with the availability of high-resolution SNP-arrays containing both copy number and SNP probes, it is now possible to identify previously hidden CN-LOHs. Mitotic recombination between pairs of homologous chromosomes is believed to be the underlying mechanism generating CN-LOHs [32], [33]. Studies have shown that CN-LOHs tend to localize within fragile sites, previously known regions of frequent genomic instability [32], [33]. Potential clinical utility of CN-LOHs is recently being appreciated, as CN-LOHs are associated with duplication of oncogenic alleles with simultaneous loss of normal functional alleles.

We have validated three CN-LOHs in an independent genotyping platform and with the following generalized features:

- A CN-LOH at 17q11.2 showed significant associations with BCR in unstratified 363 samples while comparable log-rank *P* values and HRs (increased risk) were also observed in the molecularly stratified 208 luminal A samples. The CN-LOH also demonstrated entire overlap with multiple germline CNVs in DGV, including both copy number gains and losses. This CN-LOH harboured three known genes such as suppressor of zeste12 homolog (*Drosophila*) (*SUZ12*), leucine rich repeat containing 37B (*LRRC37B*) and SH3-domain GRB2-like 1 pseudogene 1 (*SH3GL1P1*). *SUZ12* is a zinc finger gene often found at the breakpoints of recurrent chromosomal translocation in endometrial stromal sarcoma [39]. It has also been shown to act as a transcriptional repressor of Homeo box protein Hox-A9 gene in primary breast cancers through DNA hypermethylation and recruitment of DNA methyltransferases [40]. Protein encoded by *LRRC37B* gene is not well-characterized yet. However, a recent study has reported that the *LRRC37B* locus may harbour non-allelic homologous recombination hotspot, a major mechanism involved in chromosomal rearrangements [41]. *SH3GL1P1* is a pseudogene with no known function. Chromosome 17q11.2 region is also known to harbour CNVs as this loci is a hot spot for segmental duplications [42].
- The CN-LOH was more specific to the luminal A subtype of breast cancer as log-rank *P* values and HRs were comparable in luminal A samples only but were statistically insignificant in other sub-phenotypes such as HER2 type and triple negative, except in the luminal B subtype (albeit at the limited sample size for other subtypes of breast cancer). Recently, distinct CNA profiles were reported for molecular subtypes of breast cancer [43] and the findings from our study not only support such a premise but also extend these observations to the disease outcomes. On the other hand, intrinsic molecular similarities between the luminal A and luminal B subtypes of breast cancer, especially in terms of ER and PR status, may be attributed to similar log-rank *P* values in both groups;
- The two remaining CNAs at 11q13.1 and 6q24.1 were detected in subgroup analyses restricted to luminal A cases (BCR = 80, non-BCR = 128) showing significant differences in RFS probabilities and conferred risk to BCR. Both CN-LOHs at 11q13.1 and 6q24.1 are novel and did not harbour any known genes; however, these may still influence the phenotype through cis-acting regulatory activities. CN-LOH at 11q13.1 did not contain any known genes but solute carrier family 22 (organic anion/urate transporter) (*SLC22A11*) was located ∼37.19 kb down-stream of the CNA. The integral membrane protein encoded by the *SLC22A11* gene acts as an organic anion transporter, which mainly involves in transfer of estrone 3 sulfate through plasma membrane [44]. CN-LOH at 6q24.1 also did not contain any known genes; however, microRNA 3668 (*MIR3668*) and microRNA 4465 (*MIR4465*) were found ∼105.18 kb upstream and ∼281.64 kb down-stream of this CNA. Both *MIR3668* and *MIR4465* encode microRNAs, short non-coding RNA molecules involved in post-transcriptional modifications of eukaryotic organisms.

Even though we did not perform independent survival analysis with the non luminal A molecular sub-phenotypes of breast cancer (luminal B, HER2 type and triple negative) owing to limited sample size, our results provide a rationale for conducting such analyses to identify germline CNAs specific to these molecular subgroups. Analyses based on finer classification of molecular subtypes of breast cancer encompassing ki67 marker in addition to the cell surface receptor (ER, PR and HER2 status) based classifications described here and the newly described molecular subtypes in breast cancer [43], [45], may help identify more informative germline CNAs that potentially explain larger proportion of heterogeneity in breast cancer prognosis. Clinical utility of the identified germline CNAs showing strong prognostic value will be favorable if these markers are reproduced in larger but independent studies.

**Figure 4:**
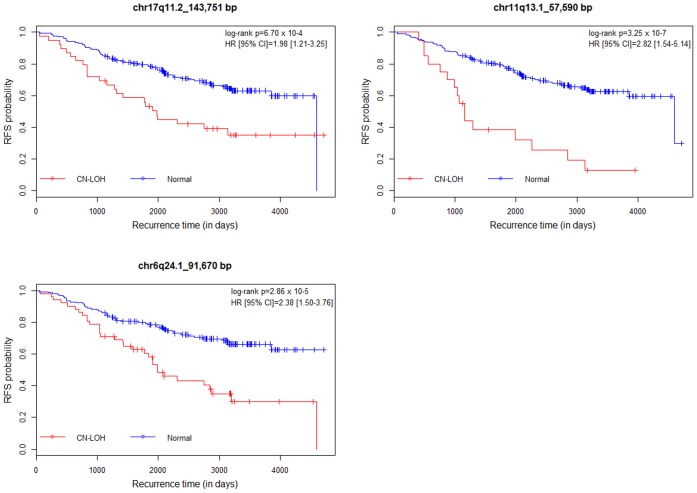
Relationships between RFS and three CN-LOHs validated by RT-qPCR and Sequenom genotyping. Using the data from 208 luminal A cases, Kaplan-Meir survival plots were generated to evaluate the predictive power of three CN-LOHs validated in independent platform for RFS. The x-axes in all three plots show recurrence time in days and the y-axes show RFS probabilities with and without CN-LOHs. Differences in RFS probabilities were assessed by log-rank tests with one d.f. HRs and 95% CIs were estimated by Cox proportional hazards model adjusted for tumor stage and grade.

In summary, we found multiple germline CNAs at chromosomes 6, 11 and 17 (results confirmed from independent genotyping platforms, Figure 4 and Table S4) with potential prognostic value, independent of tumor grade and tumor stage for early-stage non-metastatic luminal A subtype of breast cancer. Despite the large collection of recurrent cases from a single source (derived from Alberta) with extensive follow-up and outcomes data, the sample size needed for independent replication of these findings therefore warrant large international collaborations. Further investigations in to the biochemical and molecular basis for the prognostic significance of the genomic signatures may aid in the development of targeted therapeutics and molecularly driven strategies to reduce the risk of BCR.

## Supporting Information

Table S1 **Details of TaqMan copy number assays and SNPs genotyped for validation.**
(XLSX)

Table S2 **A total of 19,591 CNVs and CN-LOHs identified in entire 363 samples.**
(XLSX)

Table S3 **Relationship of top ten CNAs (** **Tables 2** **and** **4** **) with BCR in luminal B, HER2 type and triple negative subtypes of breast cancer samples.**
(XLSX)

Table S4 **Validation of three CN-LOHs in subset of 208 samples using RT-qPCR and Sequenom genotyping.**
(XLSX)
